# Micronutrient Biomarker Selection and Assay Methods and Performance in Double-Blind, Randomized, Controlled Micronutrient Dose Response (MiNDR) Trials among Women of Reproductive Age and Pregnant Women in Rural Bangladesh

**DOI:** 10.1016/j.cdnut.2025.107546

**Published:** 2025-09-01

**Authors:** Sulagna Bandyopadhyay, Anjan Kumar Roy, Sarah Baker, Katherine K. Stephenson, Ximing Ge, Yuwei Wang, Khalid Bin Ahsan, Eleonor Zavala, Hasmot Ali, Rezwanul Haque, Lee Shu Fune Wu, Brooke Langevin, Mathangi Gopalakrishnan, Towfida Jahan Siddiqua, S.M. Tafsir Hasan, Parul Christian, Kerry J Schulze

**Affiliations:** 1 Center for Human Nutrition, Department of International Health, Johns Hopkins Bloomberg School of Public Health, Baltimore, MD, United States; 2 Nutrition Research Division, International Centre for Diarrheal Disease Research, Bangladesh (icddr,b), Dhaka, Bangladesh; 3 JiVitA Project, Rangpur, Bangladesh; 4 Center for Translational Medicine, University of Maryland School of Pharmacy, Baltimore, MD, United States

**Keywords:** multiple micronutrient supplementation, vitamins, minerals, pregnancy, women of reproductive age, biomarker, analytical method

## Abstract

**Background:**

Comprehensive documentation of micronutrient biomarker assessments, capturing status from deficiency to excess, remains limited, specifically in the context of multiple micronutrient supplementation (MMS) trials.

**Objectives:**

We document biomarker selection, preanalytical and analytical methods, assay performance evaluation, and biomarker interpretation for modeling the dose–response effects of MMS in 2 parallel bioefficacy trials among women of reproductive age and pregnant women in rural Bangladesh.

**Methods:**

Blinded analysis of biomarker assays is being performed in the field and at 2 laboratories. Automated clinical chemistry analyzers are used to measure conventional serum and plasma biomarkers of vitamin D, B12, folate, iron, inflammation, iodine, and bone turnover. Plasma vitamers of A, E, B2, and B6, and urinary B1, B2, and B3 are measured by ultra-performance liquid chromatography (UPLC). A serum mineral panel is analyzed by inductively coupled plasma mass spectrometry (ICP-MS). Urinary iodine and functional assays for vitamin B1, B2, and B12, iron, and selenium are measured using 96-well plate methods. Point-of-care tests are performed for hemoglobin in venous blood, whereas liver and kidney function, glucose, and a lipid panel are performed in plasma.

**Results:**

Limits of detection and quantitation for biomarker assays are reported. Interassay coefficient of variations of quality control (QC) materials for primary outcome biomarkers are 4%–10% for automated analyzers, ICP-MS, and 96-well plate, and 2%–11% for UPLC assays, where available. Measurements of two-thirds of the primary outcome biomarkers could be evaluated using established external QC materials to ensure assay performance.

**Conclusions:**

The detailed account of micronutrient biomarker assays in the dose–response MMS trials provides a useful framework for designing future research involving comprehensive assessments of micronutrient status in vulnerable populations. External quality assurance tools are warranted for UPLC-based B1, B2, and B3 vitamers, and for kinetic assays of B1, B2, and selenium.

## Introduction

Micronutrient deficiencies remain prevalent in women living in low and middle-income countries (LMICs) [[1], [2], [3]], and are associated with adverse birth outcomes and impaired child growth and development [2,4,5]. Antenatal multiple micronutrient supplementation (MMS) trials provide robust evidence of a causal association between maternal micronutrient deficiencies and adverse pregnancy and birth outcomes [[6], [7], [8]]. The most studied formulation, the United Nations International Multiple Micronutrient Antenatal Preparation (UNIMMAP), containing 1 recommended dietary allowance (RDA) of 15 essential micronutrients, including iron and folic acid (IFA) [9], has been shown to reduce low birth weight and small-for-gestational-age (SGA) births compared with standard antenatal care with IFA alone [6]. However, most MMS trials assess the effectiveness of the interventions using maternal and infant health outcomes—such as pre-eclampsia, anemia, SGA, preterm delivery, stillbirth, mortality, and infant growth [[6], [7], [8],^10^]. Although these indicators are robust and reliable, they do not directly reflect changes in the status of supplemented micronutrients, require a longer duration to evaluate the effectiveness of interventions, and capture outcomes that are often irreversible and have lifelong consequences [11,12]. In contrast, biomarkers are more sensitive indicators of nutritional status and can serve as complementary outcome measures for detecting nutrient deficiencies at preclinical stages, and for the efficient and rapid evaluation of nutrient-specific responses to interventions [11,13], making them valuable tools for informing evidence-based nutrition interventions in public health settings [11,14,15].

Although MMS with the UNIMMAP formulation improves maternal and neonatal health [6], studies assessing micronutrient status showed that it did not fully address micronutrient deficiencies, particularly in undernourished settings [16,17]. For example, in an antenatal MMS trial in rural Bangladeshi women [17], the conventional MMS reduced the prevalence of vitamin B12, A, D, and zinc deficiencies by 15%–40% at 32 wk of gestation compared with the IFA group, but deficiencies persisted, with plasma vitamin B12 and zinc concentrations declining with gestational age [17], and limited effects on cord blood biomarkers [18]. Single- or dual-nutrient randomized controlled trials using higher antenatal dosages (>1 RDA) of vitamin D [19] and vitamin B12 [[20], [21], [22]] safely corrected deficiencies, and higher dose-MMS formulations compared with UNIMMAP [[23], [24], [25]] have shown greater benefits for birth outcomes, although effects on micronutrient status were not assessed. Additionally, most women in LMICs enter pregnancy with pre-existing deficiencies [1,17,[26], [27], [28]], yet only a few MMS trials have been conducted in nonpregnant women (PW) of reproductive age (WRA) or assessed micronutrient status biomarkers beyond hemoglobin [29]. In our previous antenatal MMS trial, 93.6% of rural Bangladeshi women entered pregnancy with ≥1 micronutrient deficiency, and 67.2% had 2 or more abnormal micronutrient indicators [17]. Together, these findings suggest that conventional MMS might not meet the increased nutrient requirements during pregnancy in undernourished settings. There is a need to study the response of micronutrient status to incremental levels of MMS in both WRA and PW to inform effective intervention strategies to resolve micronutrient deficiencies before and during pregnancy, to prevent early reproductive failure [30,31], and improve maternal and child health outcomes [[32], [33], [34]].

To address this gap, we are conducting 2 parallel, independent, double-blind, randomized, 4-arm controlled trials—micronutrient dose response (MiNDR)—to assess the effects of varying levels of 19 micronutrients in WRA and PW in rural northern Bangladesh [35]. We are measuring biomarkers that reflect a continuum of status, from deficiency to excess, to estimate the minimum amount and duration of supplementation required to achieve sufficiency without excess using pharmacokinetic and pharmacodynamic modeling approaches, and these have been detailed in the trial protocol [35]. However, as highlighted in several scientific convenings and reports [11,36,37], a comprehensive documentation of micronutrient status biomarkers, including their strengths, limitations, and applicability in field trials and programmatic settings, remains limited. This article aims to fill this gap by consolidating analytical plans for over 30 static and functional micronutrient biomarker assays in the context of MiNDR trials, representing one of the most extensive biomarker-based assessments conducted in any micronutrient supplementation study to date. The article also outlines the rationale for the selection of biomarkers, key analytical and preanalytical considerations, a list of quality control (QC) materials available for evaluating assay performances, assay characteristics based on preliminary data, and guidance on data interpretation, to offer a practical and accessible resource for researchers and program implementers conducting micronutrient intervention trials in LMICs.

## Methods

### Trial setting, population, and intervention

The MiNDR trials were conducted at the JiVitA Project site in rural Gaibandha district, located in the Rangpur Division of northwestern Bangladesh. This population site typifies rural Bangladesh [38]. An area-wide (75 community clusters, or sectors) household census was conducted to identify all married women aged 18–35 y and assess their eligibility for inclusion in pregnancy surveillance or the WRA trial, as recently described [35]. The trials were approved by the Institutional Review Board of Johns Hopkins University BSPH and the Research and Ethical Review Committees of icddr,b, Bangladesh, and were registered on ClinicalTrials.gov (NCT06081114). A Data Safety and Monitoring Board was established to review the progress of the trials.

Participants in both trials were randomly assigned to 4 study arms and supplemented with increasing levels of 19 micronutrients, ranging from 1 RDA/adequate intake up to as much as ∼13 times the recommended intakes or ≤∼75% of the tolerable upper intake level, where applicable (Supplemental Table 1). Vitamin A, D, E, B1, B2, B3, B6, B12, and selenium were administered in 4 incremental doses, with choline provided in 3 doses (compared with no choline in the control arm), and vitamins C, zinc, iron, and iodine in 2 doses. The doses for folate, vitamin K, B5, biotin, potassium, calcium, phosphorus, copper, manganese, and magnesium remained constant across the intervention arms. The daily nutrient doses were provided through a flavored powder mixed with water to reconstitute a drink, alongside a fortified balanced energy and protein (BEP) food product, containing ∼1 RDA of 18 micronutrients per serving (Supplemental Table 1). One of the flavored powders was a nutrient-free placebo drink for the control group. The supplementation period was 3 mo for WRA and ∼6 mo for PW from 12 to 16 wk of gestation until birth, and adherence was ensured by direct daily observation. The nutrient content in the micronutrient powder supplements, BEP product, and the blinding protocol were described previously [35]. Final sample size goals were 240 participants per trial, with 60 per arm.

### Selection of biomarkers to assess dose–response

Following an extensive review of the literature, biomarkers were selected, and appropriate biospecimen collection and processing plans were made [19,[39], [40], [41], [42], [43], [44]]. For example, the most responsive and appropriate biomarkers require specific sample types: erythrocyte transketolase or glutathione reductase activities for vitamin B1 and B2, respectively; urinary excretion of vitamin B1, B2, B3 vitamers, and iodine; serum or plasma is preferred for some biomarkers. Biomarkers can be static (nutrients or metabolites in circulation or excreted) or functional (reflecting a role the micronutrient plays) and can indicate recent nutrient exposure or longer term status (e.g., stores); understanding what aspects of status a biomarker reflects is crucial for precise interpretation of findings [44]. Where possible, we chose >1 indicator per nutrient to obtain complementary information on status, and the postpartum assessment was chosen to determine whether changes in status during pregnancy were sustained following cessation of supplementation. In addition to biomarkers selected for their specificity in reflecting micronutrient status, clinical biomarkers were assessed to ensure safety during the trials [35], and endocrine, inflammatory, and other ancillary biomarkers are being measured to aid in the interpretation of primary outcomes. Lastly, the reliability of assays and logistical considerations—such as laboratory capacity, instrumentation facilities, technical expertise, human resources, availability of conventional methods, and quality control/quality assurance materials —were also carefully evaluated for the selection of biomarkers and analysis.

### Preanalytical conditions: biospecimen collection, processing, storage, and transit

#### Biospecimen collection

For the WRA trial, blood and urine were collected at baseline (presupplementation), mid-line (6 wk), and at the end of supplementation (12 wk). In the PW trial, following gestational age dating via ultrasound, biospecimens (blood and urine) were collected at baseline (<16 wk of gestation), mid-pregnancy (either 22 or 28 wk of gestation), and at the end-of-supplementation (∼34 wk of gestation). Stool samples were collected from women in both trials at baseline and the end-of-supplementation visits. The final biospecimen collection visit in the PW trial occurred at 1 mo postpartum, at which time a breastmilk sample was collected, along with the blood and urine samples. Labels for biospecimens (CILS International) were designed for integration into trial workflow and preprinted on rolls with alpha-numeric identifiers and corresponding barcodes specific to each trial, visit, sample type, aliquot, and chronological identifiers, enabling each sample to be linked to a woman’s unique, randomly assigned study identifier.

All biological samples (blood, urine, feces, and breastmilk) were collected by trained data collectors at the participants’ homes following standard operating procedures. Venous blood (12 mL) was drawn from each participant into 2 evacuated tubes by phlebotomists designated as study technicians (STs): a trace element-free serum tube and a lithium heparin tube (Becton Dickinson). Blood samples were collected in the morning, typically in a fasted state, to minimize the contribution of diurnal variability to the imprecision of the biomarker assays and to ensure that biomarkers reflect steady-state conditions rather than postprandial changes; thus, on the day of biospecimen collection, the daily supplementation visit was scheduled for after blood collection. The STs tested hemoglobin in venous blood during each biospecimen collection visit by a point-of-care instrument (Hemocue 301, Hemocue), using the residual blood in the tubing attached to the needle used for blood collection.

Participants were left with supplies and instructions for self-collection of 20 mL of mid-stream urine from the first void of the day and a stool sample in DNA/RNA shield fecal collection tubes (Zymo Research Corporation; ∼1 g or 1 mL in volume, or 4 mL in case of watery stool) on the subsequent (or shortly thereafter) day. Supplies included 70 mL sterile collection cups (STARPLEX) and DNA/RNA shield fecal collection tubes (Zymo Research Corporation) to preserve both DNA and RNA for future metagenomics and transcriptomics, respectively (not further described here).

At the 1 mo postpartum visits, breastmilk samples were collected by STs through full breast expression using an electric breastmilk pump (Medela Symphony), from which ∼10 mL milk sample was transferred into sterile, trace element-free containers (Fisher Scientific) at the time of collection, and the remaining milk was returned to the participant in a sterile bowl-spoon set for spoon-feeding the infant. A day before the collection, participants were instructed not to feed their infants from 1 breast the following morning to optimize full breast expression during the sample collection.

All biospecimens were protected from light to preserve the stability of photosensitive vitamins during collection and were transported from the households to the temperature-controlled field laboratory in coolers with cold packs. All samples were processed and stored within a few hours of collection in the field, with date and timestamps recorded at each step—collection, receipt at processing laboratory, admission to storage—by a customized tracking system with barcode readers. To ensure that 240 participants completed each trial, we enrolled 289 WRA and 354 PW, resulting in over 750 samples from the WRA and 1100 samples from the PW trials, which include the 3 or 4 time points per trial after accounting for loss to follow-up.

#### Biospecimen processing

Serum was obtained after centrifuging the clotted content in the trace element-free tube at 3000 × g for 15 min at 4°C (Sorvall ST40R, Thermo Fisher Scientific) and preserved in 3 prelabeled DNAase and RNase-free 1.2 mL cryovials (Nalgene, Thermo Scientific). An aliquot of whole blood in a 1.2 mL cryovial was obtained from the lithium heparin tube before centrifuging to acquire plasma. After plasma separation (by centrifuging at 3000 × g for 15 min) and preservation in two 1.2 mL cryovials, the remaining red blood cells were washed with cold normal saline solution (0.9% NaCl), centrifuged, and supernatant decanted 3 times to obtain a red blood cell (RBC) pellet preserved in a 2 mL cryovial after discarding the buffy coat layer at the interface of the saline and RBC pellet, except at the end-of-supplementation visit when the buffy coat layer was collected at the onto paper cards (QIAcard FTA cards, Whatman) after the second wash to preserve nucleic acids for future potential analyses. The number of aliquots per sample type was planned such that no aliquot would undergo >3 freeze–thaw cycles during the analysis.

In the field laboratory, a small amount of whole blood from the lithium heparin tubes was used for duplicate hematocrit measurements, and plasma (∼200 μL) to assess clinical biomarkers using a point-of-care instrument (Piccolo Xpress, Abbott). Urine (3 × 2 mL aliquots) samples were preserved. A breastmilk creamatocrit (similar to hematocrit) measurement was also conducted at the field laboratory to estimate the lipid content of milk samples [45].

#### Biospecimen storage and transit

Following processing, the aliquots of whole blood, plasma, serum, RBC pellet, urine, and milk were stored at –80°C in the JiVitA laboratory. Buffy coat samples were stored at –20°C in a desiccator. For micronutrient biomarker analysis, samples were shipped periodically from the field laboratory in Rangpur to the Immunobiology, Nutrition, and Toxicology laboratory at icddr,b in Dhaka, Bangladesh and to Baltimore, USA to the Center for Human Nutrition Laboratory at Johns Hopkins Bloomberg School of Public Health (BSPH) using dry ice, liquid nitrogen or ice packs, depending on the sample type. On receipt, samples were stored in –80°C freezers until analysis. Stool samples collected in DNA/RNA shield collection tubes were directly stored at –20°C in the JiVitA laboratory and later shipped to the icddr,b Genome Center for metagenomic sequencing.

### Analytical plans for nutrient-specific biomarker assays

The analytical plans for primary, secondary, and ancillary biomarkers are presented in TABLE 1, TABLE 2. This section outlines selected biomarkers by nutrient, specimen types, and methodologies chosen for analysis, along with available QC materials for evaluating assay performance and sites of analysis. The tables also provide details on assay characteristics, rationale for their selection, and interpretation, which will be further elaborated in the subsequent sections.

**TABLE 1:** Primary biomarker assays for measuring response to micronutrient supplementation in MiNDR trials.

Nutrient	Biomarker	Specimen	Method	LOD/LOQ^1^	QC materials	Lab	Interpretation^2^
Vitamin A	Retinol	Plasma	UPLC-PDA	0.11/0.35 μmol/L	SRM 968f, 2 levels	BSPH	• Reflects liver stores when low; positively associated with status and intake • Deficiency, <0.7 μmol/L
	Retinyl palmitate	Plasma	UPLC-PDA	0.13/0.40 μmol/L	SRM 968f, 2 levels (consensus values)	BSPH	• Indicates hypervitaminosis-A at >10% of total vitamin A in nonpostprandial state
	RBP	Serum	ELISA	0.224 ng/mL	Manufacturer provided, 3 levels; Quantikine Immunoassay, R&D systems; in-house pools	icddr,b	• Related to retinol on a 1:1 molar basis.
Vitamin D	25(OH)D^3^^,^^4^	Serum	ECLIA	7.5/15.0 nmol/L	Manufacturer provided, 2 levels; PreciControl, Roche Diagnostics	icddr,b	• Primary biomarker of status from sunlight, food, and supplements • Deficiency, <30 nmol/L, insufficiency, <50 nmol/L, excess ≥375 nmol/L
Vitamin E	α-tocopherol; γ-tocopherol	Plasma	UPLC-PDA	α-tocopherol: 2.67/8.09 μmol/L γ-tocopherol: 2.03/6.15 μmol/L	NIST SRM 968f, 2 levels	BSPH	• α-tocopherol preferred biomarker of status; deficiency <12 μmol/L • α-tocopherol increases proportionally with circulatory lipids in pregnancy, so α-tocopherol (μmol): cholesterol (mmol) may be more meaningful, normal range 2.2–2.5 • Ratio of α-tocopherol: γ-tocopherol ∼ 10:1, with γ-tocopherol declining with improved status
Vitamin B1, thiamine	ETKa	RBC pellet	EKA	—	In-house pools	BSPH	• Thiamine-dependent functional and most useful indicator of status for at-risk populations. • Deficiency, >1.25; insufficiency, >1.15
	Thiamine excretion	Urine	UPLC-PDA	—	In-house pools	BSPH	• Excretion increases with recent intake once tissue compartments are saturated • Deficiency, <90 nmol/g creatinine; Insufficiency, <220 nmol/g creatinine
Vitamin B2, riboflavin	EGRa	RBC pellet	EKA	—	In-house pools	BSPH	• Enzymatic indicator of riboflavin status; reliable test for long-term status • Deficiency, >1.3; insufficiency, >1.2
	Urinary riboflavin (FMN, FAD, free riboflavin)	Urine	UPLC-FLR	—	In-house pools spiked with analytical standards	BSPH	• Excretion increases at tissue saturation, reflecting dietary intake and responsive to supplementation • Deficiency, <27 μg/g creatinine; insufficiency, <80 μg/g creatinine • Deficiency in pregnancy, second trimester: <23 and third trimester <21 μg/g creatinine • Insufficiency in pregnancy, second trimester: <55 and third trimester <50 μg/g creatinine
Vitamin B3, niacin	NMN, 2-PY	Urine	UPLC-PDA	—	In-house pools	BSPH	• Reliable for assessing niacin status; excretion increases at tissue saturation • NMN is more sensitive to marginal intakes than 2-PY • Deficiency, NMN: WRA and PW first trimester <0.5, second trimester <0.6, third trimester <0.8 mg/g creatinine • Deficiency, 2-PY/NMN: <0.5
Vitamin B6, pyridoxine	PLP, 4-PA	Plasma	UPLC-FLR	PLP: 0.67/2.02 nmol/L 4PA: 5.89/17.8 nmol/L	SRM 3950, 2 levels (uncertified values for 4PA)	BSPH	• PLP primary indicator of vitamin B_6_ status • Inadequacy at <20 nmol/L
Vitamin B12	Total cobalamin^3^^,^^4^	Serum	ECLIA	100/150 pg/mL	Manufacturer provided, 2 levels, Eleesys PreciControl, Roche Diagnostics	icddr,b	• Total B12 positively associated with status/intake. • Deficiency, <150 pmol/L; insufficiency, <221 pmol/L
	HoloTC	Serum	ELISA	8.1/8.3 pmol/L	Manufacturer provided, 2 levels, Tecan, IBL International GmbH; in-house pools	BSPH	• Biologically “active” form of vitamin B_12;_ likely a better indicator of B12 status; ∼20%–30% of total cobalamin • Deficiency is typically defined as <32–35 pmol/L.
	Homocysteine	Plasma	CLI	1.2/2 μmol/L	Commercial controls, 3 levels, Lyphocheck, Bio-Rad Laboratories Inc.	BSPH	• A functional but nonspecific biomarker of B12 status • Elevated in B12 deficiency, >15.0 μmol/L
Selenium	Circulatory selenium	Serum	ICP-MS	0.9 μg/L	SRM 1598a, 1 level Commercial controls, 2 levels, Seronorm Trace Elements Serum, SERO	BSPH	• Responsive to dietary intake, but depends on bioavailability of the consumed form. • Deficiency typically defined as <70 μg/L; excess: >400 μg/L
	GPX-3	Plasma	EKA	<50 nmol/min/mL	Manufacturer-provided positive control (Cayman and in-house pool)	BSPH	• Functional plasma biomarker of status; responsive to supplementation
Zinc	Circulatory zinc	Serum	ICP-MS	9.6 μg/L	SRM 1598a, 1 level Commercial controls, 2 levels, Seronorm Trace Elements Serum, SERO	BSPH	• Primary indicator of status; low serum zinc linked to low intakes; affected by diurnal variation • Deficiency: females (≥10 y), <70 μg/dL (morning, at fasting), <66 μg/dL (morning, at postprandial), and <59 μg/dL (afternoon)
Iron	Ferritin^3^^,^^4^	Serum	ECLIA	0.50 ng/mL	Manufacturer provided, 2 levels, Eleesys PreciControl, Roche Diagnostics	icddr,b	• Reflects iron stores • Elevated during inflammation • Deficiency, <15 μg/L; excess, >150 μg/L, indicates risk of iron overload
	sTfR^3^	Serum	IT	0.40/0.50 ng/mL	Manufacturer provided, 2 levels, sTfR control set, Roche Diagnostics	icddr,b	• Indicator of tissue iron demand. • Deficiency, >8.3 mg/L
Iodine	Urinary iodine^5^	Urine	Colorimetric	2/5 μg/L	Established controls, 2 levels, EQUIP, CDC	icddr,b	• Reflects recent exposure at population level • Cutoffs defined by median concentrations: WRA—severe deficiency <20 μg/L, moderate 20–49 μg/L, mild 50–99 μg/L, adequacy 100–199 μg/L; risk of excess 200–299 μg/L, excess >300 μg/L. • Pregnancy: deficiency <150 μg/L, excess ≥500 μg/L
	Thyroglobulin	Plasma	CLI	0.2/0.9 ng/mL	Commercial controls, 3 levels, lyphocheck tumor marker control, Bio-Rad Laboratories Inc.	BSPH	• Functional indicator of long-term iodine status, suitable for assessing individual-level status. • Elevated in iodine deficiency, typically defined as ≥13 μg/L

Abbreviations: CDC, Centers for Disease Control and Prevention; CLI, chemiluminescent immunoassay; ECLIA, electrochemiluminescent immunoassay; EGRa, erythrocyte glutathione reductase activity; EQUIP, Ensuring the Quality of Urinary Iodine Procedures; ETKa, erythrocyte transketolase activity; FAD, flavin adenine dinucleotide; FMN, flavin mononucleotide; GPX, glutathione peroxidase; Holo-TC, holotranscobalamin; 25(OH)D, 25-hydroxy vitamin D; icddr,b, International Centre for Diarrheal Disease Research, Bangladesh; ICP-MS, inductively coupled plasma mass spectrometry; IT, immunoturbidimetric; LOD, limit of detection; LOQ, limit of quantitation; NA, not applicable; NIST, National Institute of Standards and Technology; NMN, N'-Methylnicotinamide; 2PY, N-methyl-2-pyridone-5-carboxamide; PLP, pyridoxal 5’-phosphate; 4-PA, 4-pyridoxic acid; QC, quality control; RBC, red blood cell; RBP, retinol binding protein; sTfR, soluble transferrin receptor; SRM, standard reference material; UPLC-FLR, ultra-performance liquid chromatography-fluorescence detector; UPLC-PDA, ultra-performance liquid chromatography-photodiode array detector.

1 Values are reported as LOD/LOQ; only LOD are reported where LOQ are not available. Denoted as “—” for assays currently undergoing standardization.

2 Refer to the nutrient-specific discussion for the rationale and citations supporting the selected cutoff values for the micronutrient biomarkers.

3 Assay performance evaluated through external quality assurance programs—vitamin A laboratory—external quality assurance (VITAL-EQA) [48], and micronutrients performance verification (MPV) programs by CDC [49].

4 Assay performance evaluated through the Laboratory Accreditation Program by the College of American Pathologists program (CAP) [57].

5 Assay performance evaluated through external quality assurance program– EQUIP by CDC [63].

**TABLE 2:** Biomarker assays for safety evaluation, secondary outcomes, and required for interpretation of primary outcomes in MiNDR trials.

Biomarkers	Specimen	Method	LOD/LOQ^1^	QC plan	Lab	Interpretation^2^
Clinical biomarkers						
Hemoglobin (Hb)	Whole blood	Hemocue 301	Measurement range: 0–25.6 g/dL	Factory calibrated	JiVitA	• Biomarker of anemia • Severity is defined by Hb cutoffs; WRA—mild: 110–119, moderate: 80–109, severe: <80 g/L. • Trimester-specific cutoffs are provided during pregnancy; first trimester—mild: 100–109, moderate: 70–99, severe: <70 g/L; second trimester—mild: 95–104, moderate: 70–94, severe: <70 g/L; third trimester—mild: 100–109, moderate: 70–99, severe: <70 g/L
General Biochemistry panel—calcium (Ca), potassium (K), sodium (Na), chloride (Cl), creatinine, glucose, blood urea nitrogen (BUN), total carbon dioxide	Plasma	Piccolo Xpress, Basic metabolic panel	Ca: 4.0 mg/dL K: 1.5 mmol/L Na: 110 mmol/L Cl: 80 mmol/L Creatinine: 0.2 mg/dL Glucose: 10 mg/dL BUN: 2.0 mg/dL Total carbon dioxide: 5 mmol/L	Internal self-check; manufacturer-provided control, 1 level (Randox Laboratories)	JiVitA	• Acute or chronic kidney dysfunction (creatinine, >1.1 mg/dL; BUN >20 mg/dL) • Hypo/hypercalcemia (Ca, <8.8 or >10.4 mg/dL) • Hypo/hypernatremia (Na, <136 or >145 mmol/L) • Hypo/hyperkalemia (K, <3.6 or >5 mmol/L) • Metabolic/respiratory acidosis (total carbon dioxide, <20 mmol/L), or alkalosis (>29 mmol/L) • Hyperglycemia (random blood glucose, >200 mg/dL)
Lipid panel^3^—TG, TC, HDL, LDL, VLDL, liver enzymes—ALT and AST	Plasma	Piccolo Xpress, lipid panel Plus	TG: 20 mg/dL TC: 20 mg/dL HDL: 15 mg/dL ALT: 5 U/L AST: 5 U/L	Internal self-check; manufacturer-provided control, 1 level	JiVitA	• Abnormal liver enzymes (ALT, >60 IU/L, AST, >46 IU/L), and risk of metabolic syndrome, (triglyceride >150 mg/dL, total cholesterol >200 mg/dL, HDL <40 mg/dL, LDL >100 mg/dL) • Total cholesterol values will be used to interpret α-tocopherol values during pregnancy (see Table 1, vitamin E)
Urinary creatinine	Urine	Colorimetric assay	—	Manufacturer provided control, Cayman Chemical	BSPH	• Used for normalizing excretion rates of urinary metabolites of vitamin B1, B2, and B3, and deoxypyridinoline
Plasma riboflavin status						
FAD, FMN, riboflavin	Plasma	UPLC	FAD: 1.72/5.22 nmol/L FMN: 3.78/11.45 nmol/L Riboflavin: 1.01/3.05 nmol/L	In-house pools spiked with analytical standards	BSPH	• Relationship with status is unclear and shows high variability
Folate status						
Folate^4^^,^^5^	Serum	ECLIA	1.2/2.0 ng/mL	Manufacturer provided, 2 levels; Eleesys PreciControl, Roche Diagnostics; In-house pools	icddr,b	• Reflects recent dietary intake and short-term folate status of individuals. • High day-to-day variability • Deficiency, <10 nmol/L
	Whole blood	CLI	—	Commercial control, 3 levels; Lypocheck whole blood control, Bio-Rad Laboratories Inc.	BSPH	• Reflects tissue folate stores and long-term status; a reliable measure of folate status. • Deficiency, erythrocyte folate <340 nmol/L
Hematocrit	Whole blood	Hematocrit reader	—	—	JiVitA	• Packed cell volume is used to estimate erythrocyte folate concentration from whole blood folate.
Inflammatory biomarkers						
AGP	Serum	IT	10 mg/dL	Manufacturer provided, 2 levels; PreciControl, Roche Diagnostics; In-house pools	icddr,b	• Indicates chronic or prolonged inflammation, >1 g/L
CRP^5^	Plasma	CLI	0.1/0.2 mg/L by manufacturer	Commercial control, 2 levels; Liquichek, Bio-Rad Laboratories Inc.	BSPH	• Reflects acute inflammation, >5 mg/L
Mineral panel						
Mineral panel: iron (Fe), copper (Cu), calcium (Ca), potassium (K), phosphorus (P), magnesium (Mg), manganese (Mn)	Serum	ICP-MS	Fe: 40.1 μg/L Cu: 0.8 μg/L Ca: 0.8 mg/L K: 0.2 mg/L P: 0.6 mg/L Mg: 0.03 mg/L Mn: method optimization ongoing	Commercial controls, 2 levels, Seronorm Trace Elements Serum	BSPH	• Some respond to dietary intakes and interventions; some are homeostatically controlled (e.g., calcium).
Hormonal biomarkers						
PTH	Serum	CLI	3.0 pg/mL	Commercial control, 3 levels; Lypocheck, Bio-Rad Laboratories Inc.	BSPH	• Functional index of vitamin D status in normocalcemic state; inversely associated with serum 25(OH)D level. • Normal range: 10–55 pg/mL.
EPO	Plasma	ELISA	<0.67 mIU/mL	Commercial control, 3 levels; Lypocheck, Bio-Rad Laboratories Inc.	BSPH	• Signals the target cells in the bone marrow to maintain or stimulate erythropoiesis, EPO rises in iron deficiency anemia. • Typically ranges between 6 and 10.6 IU/L.
Hepcidin	Plasma	Competitive ELISA	0.304/1.149 ng/mL	Manufacturer provided controls, 2 levels; DRG International Inc.	BSPH	• Regulates iron homeostasis; signals to up/downregulate iron uptake and distribution of iron. • Levels increase with the repletion of iron stores, preventing iron overload.
Bone turnover biomarker						
Pyrilinks D (deoxypyridinoline)	Urine	CLI	6 nM	Commercial control, 2 levels; IMMULITE Pyrilinks-D, Siemens	BSPH	• Bone resorption biomarker, normalized by creatinine • Typically range between 3 and 7.4 nM/mM creatinine in women.

Abbreviations: AGP, α-1 acid glycoprotein; ALT, alanine aminotransferase; AST, aspartate aminotransferase; BSPH, Johns Hopkins Bloomberg School of Public Health; CDC, Centers for Disease Control and Prevention; CLI, chemiluminescent immunoassay; CRP, C-reactive protein; ECLIA, electrochemiluminescent immunoassay; EPO, erythropoietin; FAD, flavin adenine dinucleotide; FMN, flavin mononucleotide; icddr,b, International Centre for Diarrheal Disease Research, Bangladesh; ICP-MS, inductively coupled plasma mass spectrometry; IT, immunoturbidimetric; LOD, limit of detection; LOQ, limit of quantitation; PTH, parathyroid hormone; TC, total cholesterol; TG, triglyceride.

1 Values are reported as LOD/LOQ; only LOD are reported where LOQ are not available. Denoted as “—” for assays currently undergoing standardization.

2 Refer to the nutrient-specific discussion for the rationale and citations supporting the selected cutoff values for the micronutrient biomarkers.

3 LDL and VLDL are calculated by Piccolo Xpress analyzer using total cholesterol, HDL, and triglyceride values.

4 Assay performance evaluated through external quality assurance programs—vitamin A laboratory—external quality assurance (VITAL-EQA) [46], and micronutrients performance verification (MPV) programs by CDC [47].

5 Assay performance evaluated through the Laboratory Accreditation Program by the College of American Pathologists program (CAP) [48].

Although point-of-care biomarkers were assessed at the time of sample collection in the JiVitA field laboratory, as described, other conventional micronutrient biomarker assays for vitamin A, D, B12, folate, iron, and iodine are being conducted at icddr,b. Serum 25(OH)D, ferritin, serum folate, and total cobalamin, are analyzed simultaneously using the automated immunochemistry analyzer Cobas e601 (Roche Diagnostics GmbH), and serum soluble transferrin receptor (sTfR) and α-1 acid glycoprotein (AGP) with the clinical chemistry analyzer Cobas c311 (Roche Diagnostics GmbH).

Less conventional assays—such as ultra-performance liquid chromatography (UPLC)-based assays for vitamins A, E, B1, B2, B3, and B6, inductively coupled plasma mass spectrometry (ICP-MS)-based mineral panels, functional biomarkers, and enzyme kinetic assays (EKA; for vitamin B1, B2, and selenium)—will be performed at BSPH. For most assays, particularly where manual preprocessing steps are required, complete serial sets of samples for a participant are prioritized for analysis within assay runs to limit the contribution of interassay variability to the dose–response patterns observed in the analytic phase.

#### Vitamin A and E

Circulating retinol, retinyl palmitate, α- and γ-tocopherol are extracted from plasma using a liquid–liquid hexane-based method and are measured by UPLC-photo diode array detector (UPLC-PDA, ACQUITY H-Class, Waters Corporation) [46]. Retinol and α-tocopherol serve as the primary indicators of vitamin A and E status, respectively, whereas retinyl palmitate is an indicator of excess vitamin A [42]. Human serum-based standard reference material (SRM) 968f from the National Institute of Standards and Technology (NIST), providing certified values for retinol, α- and γ-tocopherol, and a consensus value for retinyl palmitate, was initially used to establish the validity of the assay and is also assessed with each sample run. The ratio of α-tocopherol to total cholesterol is often regarded as a more reliable indicator of vitamin E status, particularly during pregnancy [47]; thus, total cholesterol measured using the Piccolo Xpress point-of-care instrument will be used to express vitamin E status in this way. Serum retinol binding protein (RBP) is measured using a sandwich ELISA (Human RBP4 ELISA Kit, Quantikine Immunoassay, R&D Systems, Inc.) as a proxy measure of vitamin A status. Tri-level certified controls serve as QC materials for RBP measurements.

#### Vitamin D

Serum 25(OH)D, the major circulating form of the vitamin, is measured by an automated competitive binding electrochemiluminescent immunoassay (ECLIA; Cobas e601, Roche Diagnostics). This assay performance is monitored through the vitamin A laboratory—external quality assurance (VITAL-EQA) program [46] and the micronutrients performance verification (MPV) program [47] by the US Centers for Disease Control and Prevention (CDC). As an ancillary biomarker of vitamin D status, serum parathyroid hormone (PTH) is measured by CLI (IMMULITE 2000, Siemens). Circulating calcium, to monitor for evidence of hypercalcemia, is assessed as part of a metabolic panel (Piccolo Xpress, Abbott) and, among other minerals measured by ICP-MS (Model 7850; Agilent) in serum. The performance of all assays is evaluated using commercial QC materials, as detailed in the Tables.

#### Vitamin B1 (thiamine)

The functional measure of thiamine status, erythrocyte transketolase activity (ETKa),will be assessed in 96-well plates based on the protocol by Jones et al. [50,51]. The ETKa is expressed as a ratio between the activated/stimulated ETK activity with the addition of exogenous excess thiamine diphosphate and the basal activity of ETK [52,53]. Urinary excretion of thiamine will be analyzed by UPLC using PDA detection (ACQUITY Premier System UPLC, Waters Corporation) [52]. As reference materials are unavailable for both assays, in-house pools of each relevant sample matrix will be used as a QC method.

#### Vitamin B2 (riboflavin) and B6 (pyridoxine)

The measurement of erythrocyte glutathione reductase activity (EGRa) is a preferred method for evaluating vitamin B2 status, with a protocol recently published by Parkington et al. [53]. The ratio of stimulated EGRa with excess flavin dinucleotide (FAD) coenzyme to the basal (endogenous) activity provides EGRa coefficient, a functional indicator of vitamin B2 status. In-house pools will be produced, spiked with standards to create multiple levels of QC materials.

Plasma riboflavin, FAD, flavin mononucleotide (FMN), B6 vitamers such as pyridoxal 5’-phosphate (PLP), the primary indicator of status, and 4-pyridoxic acid (4-PA), a breakdown product of the vitamin, are simultaneously measured by UPLC with a fluorescence detector (UPLC-FLR, ACQUITY Premier System UPLC, Waters Corporation) using an aqueous extraction method. Riboflavin, FAD, FMN, PLP, and 4-PA extracted from plasma samples (45 μL) are subjected to a 15 min incubation at 65°C to prevent hydrolysis, followed by an extraction in a 200 μL aqueous solution, with internal standard, trichloroacetic acid, and acetonitrile. Extracts are then centrifuged and injected directly onto the UPLC (ACQUITY Premier-System, Waters), separated with a Waters ACQUITY Premier HSS T3 1.8μm VanGuard FIT 2.1×50 mm column, and eluted using a 10 mM ammonium formate buffer, pH 4.0, and acetonitrile gradient, over 6 min. The mobile phase is amended with 5 g/L sodium bisulfate as a derivatization agent for fluorescence emission enhancement of PLP [54]. Serum-based SRM 3950 (NIST, Gaithersburg) with certified values for PLP and “values of potential interest” for 4-PA serve as calibration tools and QC materials for the UPLC-based assay, and also as a serum pool for assessing precision of the riboflavin vitamers. Urinary excretion of riboflavin as an indication of levels beyond those needed to ensure riboflavin adequacy will be assessed along with urinary thiamine and B3 excretion products [55].

#### Vitamin B3 (niacin)

Urinary metabolites of niacin, N'-Methylnicotinamide (NMN), and N-methyl-2-pyridone-5-carboxamide (2-PY) will be measured using UPLC-PDA (ACQUITY Premier System UPLC, Waters Corporation). The concentrations of the NMN and 2-PY, relative to urinary creatinine, are sensitive and specific measures of vitamin B3 status [56], although QC products are not available for these B3 analytes. Urinary creatinine is measured by a commercially available colorimetric assay (e.g., Creatinine (urinary) Colorimetric Assay Kit, Cayman Chemical) to express these urinary analytes.

#### Vitamin B12 (cobalamin)

Serum total cobalamin (vitamin B12) is measured by automated competitive binding ECLIA (Cobas e601, Roche Diagnostics). The assay performance is monitored through the VITAL-EQA and MPV program by the CDC [46,47] and the College of American Pathologists program (CAP) [48], along with commercially available QC products analyzed per run. The biologically “active” fraction of total cobalamin bound to the transcobalamin carrier protein, thus denoted holotranscobalamin (holoTC), is quantified by commercial kit ELISA (Tecan, IBL International GmbH), validated against competitive binding radioimmunoassay technique [[58], [59], [60]]. Homocysteine, a nonspecific biomarker of vitamin B12 status, is measured by CLI (IMMULITE 2000).

#### Selenium and zinc

Serum selenium and zinc are measured by ICP-MS (7850; Agilent) as a part of a more comprehensive panel that measures 9 minerals simultaneously, including iron, copper, calcium, phosphorus, potassium, magnesium, and manganese. Serum (100 μL) is diluted with 1.6 mL of a basic diluent consisting of 4% 1-butanol, 0.15% triton X-100, 0.15% ethylenediaminetetraacetic acid, and 1% ammonium hydroxide, in liquid chromatography-mass spectrometry (LC/MS)-grade water (Fisher Scientific). An internal standard of 0.06% (100 ppm of 6-Li, Sc, Ge, Rh, In, Tb, Lu, Bi in 10% HNO_3_; Agilent) and LC/MS-grade water are added to obtain a total volume of 4 mL for analysis. Inorganic standards are used to generate standard curves, and serum-based SRM 1598a (NIST) with a certified concentration of minerals is used to monitor assay performance. The serum-based reference material (Seronorm, SERO) is analyzed with each batch of samples as quality control to check the precision of the assay. Glutathione peroxidase (GPX) activity, the functional indicator of selenium status, is measured by a commercially available EKA kit (Cayman).

#### Iron

Serum ferritin, a key biomarker of iron stores, is measured by CLI (Cobas e601, Roche Diagnostics), and sTfR with an immunoturbidimetric (IT) method (Cobas c311, Roche Diagnostics). These assays undergo annual performance evaluation through the VITAL-EQA and MPV program by CDC [46,47], and the performance of serum ferritin assay is also evaluated by the CAP program [48]. Bilevel commercial products (Eleesys PreciControl Varia Level 1 and 2, Roche Diagnostics) serve as QC materials for these assays. Hemoglobin is measured using the point-of-care Hemocue 301 (Hemocue) in venous blood. Hepcidin (Hepcidin 25 HS ELISA RUO, DRG International), with comparability to reference standard assays recently demonstrated [61], and erythropoietin (EPO; ClinMax Human EPO ELISA Kit, PRO, ACRODiagnostics Inc.), biomarkers related to iron regulation, are measured by ELISA. Serum iron is obtained from the mineral panel assessment just described.

#### Iodine

Urinary iodine is measured by a microplate method based on a modified Sandell-Kolthoff reaction [62]. A customized sealing cassette is used during this digestion process to prevent vapor loss and cross-contamination between wells. The performance of this assay is evaluated quarterly by the CDC through the standardization program, ensuring the quality of iodine procedures (EQUIP) [49]. Tri-level EQUIP controls serve as QC materials. Additionally, spike-recovery assessments with standard iodine solutions are conducted for each batch of samples. Plasma thyroglobulin is measured by chemiluminescent immunoassay (IMMULITE 2000) with commercial QC materials.

#### Secondary outcomes and biomarkers of safety evaluation

The biomarkers for nutrients that did not vary across the 4 arms of the trial (e.g., folate) are considered secondary and are detailed in Table 2, as are ancillary biomarkers, some of which were previously described. Serum folate (at icddr,b) is measured by ECLIA (Cobas e601, Roche Diagnostics), and whole blood folate (at BSPH) by automated chemiluminescence competitive binding immunoassay (IMMULITE 2000). To calculate RBC folate from whole blood folate requires hematocrit values to account for the proportionate contribution of RBCs in whole blood [64]. The performance of the serum folate assay is monitored through the CAP program [48], and commercially available controls are available for both serum folate (Eleesys PreciControl Varia level 1 and 2, Roche Diagnostic) and whole blood folate (Lypocheck whole blood control, levels 1, 2, and 3, Bio-Rad Laboratories Inc.).

Plasma C-reactive protein (CRP) by CLI (IMMULITE 2000) and AGP by IT (Cobas c311, Roche Diagnostics) are assessed as biomarkers of acute and prolonged inflammatory responses, respectively. The bone turnover biomarker, urinary deoxypyridinoline (Pyrilinks-D), is measured by CLI (IMMULITE 2000).

Clinical biomarkers are assessed using the lipid plus [65] and metabolic panel [66] by Piccolo Xpress, per manufacturer instructions, and as previously described, with monthly QC runs (Abaxis chemistry control level 1, Randox Laboratories).

A detailed description of methods for human milk and fecal samples will be provided in separate publications.

#### Evaluation of assay performance and characteristics

Based on progress to date, the limit of detection (LOD) and/or limit of quantitation (LOQ) are reported, either taken from the manufacturers’ documentation or determined within participating laboratories with calculations inherent in instrumentation software (e.g., ICP-MS) or by manual calculation for UPLC-based assays (TABLE 1, TABLE 2). The interassay coefficient of variation (CV%) for the primary outcome biomarker assays is evaluated using QC materials at different concentrations, measured in duplicates across 5 d, alongside the initial runs of MiNDR samples (Supplemental Table 2). The CV is calculated as the SD of the 5 daily mean QC values divided by the mean concentration, expressed as a percentage. We also summarize external quality assurance schemes per biomarker.

## Results

The initial analysis demonstrates that the interassay CV of the QC materials for the automated assays (25[OH]D, total cobalamin, ferritin, sTfR, folate, CRP, AGP, PTH) ranges from 4% to <10%. UPLC-based assays for primary outcome biomarkers (retinol, α- and γ tocopherol, PLP), which require multistep preprocessing procedures, demonstrate a CV of 2% to 11% depending on the analyte and the extraction process. Retinyl palmitate, a biomarker for excess vitamin A, shows a relatively higher CV (17.1%) for the QC materials at low concentration compared with the higher concentration (CV: 8.9%). The interassay variability for ICP-MS-based mineral assays (zinc, selenium, iron, copper, calcium, phosphorus, potassium, and magnesium) and ELISA or microplate-based assays (urinary iodine, holoTC, GPX) is observed as <10%. The interassay CVs for primary outcome biomarkers, which should be considered preliminary, are presented in Supplemental Table 2. Performance characteristics of some assays, such as urinary excretion of B vitamins and EKA, have not yet been established. Further details on these biomarkers will be provided in future publications.

For the primary outcome biomarkers in the MiNDR trials (Table 1), we are assessing 25 individual analytes. Among these, processes like external quality assessment programs by the CDC [46,47,49] and CAP [48], or products like SRMs or commercially available QC materials with independently established values for the analytes of interest are available for 15 (60% of all) analytes (retinol, retinyl palmitate, 25[OH]D, α- and γ-tocopherol, PLP, 4-PA, total cobalamin, homocysteine, ferritin, sTfR, serum selenium, serum zinc, urinary iodine, and thyroglobulin), suggesting reasonable tools are available to establish validity or interlaboratory comparability of vitamin A, D, E, B6, B12, iron, selenium, zinc, and iodine status. External quality assurance tools for thiamine, riboflavin, and niacin vitamers in circulation, urine, and via functional assays using erythrocytes are lacking, although specific protocols have been published for the EKA for thiamin and riboflavin, as noted. The performance of additional primary outcome assays—RBP, holoTC, and GPX activity is evaluated by the manufacturer-provided QC materials and house pools. Six analytes assessed by UPLC utilize NIST-SRMs (968f, 3950) with certified and consensus values to ensure assay validity (retinol, retinyl palmitate, α- and γ-tocopherol, PLP, and 4-PA), and NIST SRM (1598a) and Seronorm for ICP-MS mineral assays, including serum zinc and selenium as primary outcome analytes. For 2 analytes (homocysteine and thyroglobulin), commercial QC materials are available. For urinary excretion assays, where standard QCs are unavailable, such as for thiamine, riboflavin, flavin metabolites, 1-MN, and 2PY, a urine house pool spiked with analytical standards will be used. For the 2 erythrocyte EKA (ETKa, and EGRa), washed erythrocyte house pools are planned for use as QCs.

## Discussion

Comprehensive assessment of micronutrient status among vulnerable women could improve interventions to address micronutrient deficiencies before and during pregnancy. Yet remarkably little is known about women’s status, as tools and techniques for accurate assessment across a broad array of micronutrients are lacking. We summarized the selection criteria, methods, and performance characteristics for biomarker assays used in 2 trials of micronutrient interventions, and greater detail on biomarker interpretation in the context of trial outcomes is elaborated here.

### Fat-soluble vitamins

Circulating retinol reflects liver stores of vitamin A in deficiency states. A cutoff of <0.7 μmol/L is commonly used to define vitamin A deficiency [42,67]. Circulating retinol decreases during pregnancy, especially in the third trimester, due to increased demand, placental transfer, and plasma volume expansion [42,68,69]. Retinol circulates in a ∼1:1 ratio with RBP at saturated liver stores, supporting the use of the same deficiency cutoff for RBP (<0.7 μmol/L) [42], although the assay for RBP has fewer tools for global standardization, and apo-RBP can also exist in circulation [42]. Fasting elevated retinyl esters (>10% of total circulating vitamin A) indicate excess vitamin A [70], although expectations during pregnancy are unknown. Hypervitaminosis-A has been associated with bone deformities and fractures [70,71]; thus, Pyrilinks-D, a bone turnover marker [72], will be used along with liver enzymes (alanine aminotransferase and aspartate aminotransferase) as proxy and supporting biomarkers to confirm safety.

Serum 25(OH)D is the primary biomarker of vitamin D status, reflecting vitamin D exposure from diet, supplements, and sunlight—with deficiency defined as 25(OH)D <30 nmol/L, insufficiency <50 nmol/L, and excess at ≥375 nmol/L [73,74]. PTH is inversely associated with 25(OH)D, particularly when low calcium intake or absorption—secondary to inadequate vitamin D status—exists, and contributes to bone resorption [75,76]. Hypercalcemia is being monitored as it can occur with excess vitamin D exposure [73,77], although toxicity or confirmed hypercalcemia was not observed in a trial of vitamin D3 supplementation conducted among Bangladeshi women who received up the 28,000 IU weekly, which exceeded our maximum dose [19].

α-Tocopherol, the most abundant form of vitamin E in human tissues, is used to assess vitamin E deficiency [51] (α-tocopherol <12 μmol/L) [78]. As pregnancy advances, α-tocopherol increases in proportion to circulating lipids, so the α-tocopherol to plasma cholesterol ratio could be of greater use during pregnancy [51]. The other isomer of vitamin E, γ-tocopherol, is usually ∼10 times lower than α-tocopherol due to preferential retention of α-tocopherol and catabolism of γ-tocopherol [51,79]. Both isomers of vitamin E respond to supplementation, although α-tocopherol increases and γ-tocopherol decreases [17,51,79].

### Water-soluble vitamins

The assessment of EKAs, such as ETK and EGR activity as functional indices of long-term vitamin B1 and B2 status, reflects tissue stores [52,53,80], and are responsive to supplementation in deficient populations [53,80,81]. Activity coefficients—ETKac and EGRac—indicate adequacy when close to 1.0, but increase with deficiency; cutoffs of >1.25 for vitamin B1 [44,53] and >1.3 for vitamin B2 [[82], [83], [84], [85]] are commonly used. Urinary vitamin B1 and B2 excretion, expressed relative to creatinine if in a spot sample, also serve as useful short-term biomarkers of intake [44,[86], [87], [88], [89]]. During pregnancy, vitamin B1 is preferentially transferred across the placenta with severe deficiency linked to infantile beriberi, postpartum neuropathy, and gastric beriberi [[90], [91], [92]]. Demand for vitamin B2 also rises during pregnancy, particularly in the third trimester [93,94]. In the MiNDR study, plasma riboflavin and flavin metabolites are used as auxiliary biomarkers due to the lack of a well-established relationship with vitamin B2 status [80].

Urinary metabolites NMN and 2-PY are major excretory products of vitamin B3 metabolism and are considered the most reliable and sensitive biomarkers for assessing vitamin B3 intake [44,95,96]. About 20%–35% of nicotinic acid is excreted as NMN and ∼45%–60% as 2-PY [97], and 24-h urinary excretion of these metabolites correlates with dietary vitamin B3 intake [98,99]. Studies in clinical pellagra patients show lower urinary NMN and 2-PY concentrations compared with healthy controls [58,100]. Urinary metabolites are usually expressed relative to creatinine and as 2-PY:NMN to define niacin deficiency [101].

For vitamin B6, PLP is the key indicator of status [94,102], correlating with vitamin B6 intake, urinary B6 excretion, and total plasma vitamin B6 concentration [94,103,104]. Low plasma PLP is associated with impaired neurological and immunological indicators [94,[105], [106], [107]], and a cutoff <20 nmol/L is commonly used for deficiency [107,108]. Very high intakes of vitamin B6 (>200 mg/d) have been linked to peripheral neuropathy and tingling, burning, or numbness of hands or toes [94,109]. 4-PA, the main vitamin B6 metabolite, correlates positively with vitamin B6 supplementation and complements PLP as a biomarker of vitamin B6 status [110,111]. Circulating B6 vitamers decline over pregnancy, especially in the third trimester [44,94,112], likely due to plasma volume expansion [113,114] and placental transfer to the fetus [112,115].

Serum total cobalamin (vitamin B12) is the most common biomarker of vitamin B12, reflecting intake, long-term status, and liver stores [39]. Serum vitamin B12 <150 pmol/L indicates deficiency, and <221 pmol/L insufficiency [39,94]. Yet, total B12 has limited diagnostic value as a stand-alone marker [39,116], particularly in pregnancy, when serum B12 declines 25%–30% due to hemodilution and active fetal transfer [39,94,117], requiring large doses to resolve apparent B12 deficiency [[20], [21], [22]], but with potential downregulation of absorption at high intakes [118]. Serum holo-TC, the biologically active fraction of circulating B12 (5%–20% of total cobalamin), is typically affected by physiological changes during pregnancy and could be more reliable for studying the supplementation effects during pregnancy [39,59,117,119,120]. Although there is no consensus on deficiency cutoffs for holo-TC, <32 pmol/L showed good sensitivity and specificity against methylmalonic acid (MMA), a specific functional indicator of vitamin B12 status [121], although MMA is less useful in populations lacking B12 deficiency [39]. Homocysteine is another nonspecific biomarker of B12 status, as it is also increased in folate, vitamin B2, and B6 deficiencies [122]. A composite index using 2–4 vitamin B12 biomarkers could offer a more holistic assessment of status [116,123,124], but needs further validation across physiological stages and populations.

In the MiNDR trials, folate status is considered a secondary outcome since the supplementation doses were constant across the intervention arms. For efficiency of throughput and to allow for calculation of both serum and erythrocyte folate, total folate was measured in serum and whole blood by immunoassay, as opposed to techniques (microbiological, and mass spectrometry methods) that would allow for distinction of specific folate vitamers. Serum folate reflects recent folate intake, and shows high within-subject variability [40,125,126], while erythrocyte folate reflects long-term status and tissue stores [40], providing a comprehensive assessment of folate status.

### Minerals

In the MiNDR trials, among the minerals, only selenium is tested at 4 escalating doses, whereas zinc, iron, and iodine are evaluated at 2. Serum copper, phosphorus, potassium, magnesium, and manganese are part of the mineral panel but are considered ancillary biomarkers, as doses of these nutrients remain constant across intervention arms.

Serum or plasma selenium is commonly used to assess selenium status, as it reflects dietary intake or supplementation—more so with organic than inorganic sources [[127], [128], [129]]. Circulating selenium <70 μg/L is often used to define selenium deficiency in adults [130], with >400 μg/L indicating potential toxicity [131,132]. A decline in circulating selenium in late pregnancy suggests demand for maternal needs and fetal transfer [[133], [134], [135]]. Selenium is incorporated into proteins as selenocysteine, including GPX, and plays a role in antioxidant defense [127,136]. Plasma GPX-3 activity increases with total circulating selenium but plateaus as sufficiency is achieved [127,136,137].

Serum zinc is the established biomarker for evaluating population-level zinc status [43,138]. It represents only 0.1% of the total body zinc, and is tightly regulated, making it unreliable for individual-level assessments [43,[138], [139], [140]]. A population prevalence of low serum zinc ≥20% indicates a public health concern [43,138]. Specific deficiency cutoffs are also defined by age, sex, fasting, and diurnal patterns [141,142]. A meta-analysis of zinc supplementation studies showed only modest changes in serum zinc across populations [140,143,144], including a small 3% increase during pregnancy [144]. Serum zinc declines regardless of intake due to hormonal changes and plasma volume expansion in pregnancy [145]; however, cut-offs to define deficiency or insufficiency during pregnancy are unavailable. Functional biomarkers of zinc status remain underdeveloped, and stable isotope-based kinetic studies are not feasible for routine use [43].

Hemoglobin is a functional but nonspecific point-of-care biomarker for iron deficiency, as it is affected by other micronutrient deficiencies (e.g., vitamin A, B12, folate) [41] and non-nutritional conditions [146]. Given the low prevalence of iron deficiency in rural Bangladesh, likely due to high groundwater iron levels [146], we are relying on ferritin, a robust biomarker of iron status [41], to determine response to 2 levels of supplementation doses (30 and 40 mg/d). Ferritin cutoffs <30 and <15 μg/L indicate moderate to severe depletion of iron stores, whereas >150 μg/L suggests iron overload in women [147,148], and potential adjustments for inflammation are performed, if necessary [147,149,150]. During pregnancy, serum ferritin concentration declines, reaching its nadir in the third trimester, possibly due to hemodilution, increased metabolic demand, and fetoplacental development and iron transfer [151]. sTfR, the solubilized form of the cellular transferrin receptor molecule, is measured as a complementary biomarker, indicating tissue iron demand, but may have limited utility in iron-sufficient populations [41,152,153]. To further understand iron regulation in this setting, we are measuring hepcidin (downregulates iron absorption in sufficient status) and EPO (promotes erythropoiesis) in the MiNDR trials [154,155].

Median urinary iodine is a population-level indicator of iodine status, and is responsive to dietary iodine intake, with ∼90% of dietary iodine being excreted through urine [156,157]. Urinary iodine is interpreted relative to established cutoffs for PW and non-PW [158]. Thyroglobulin, which reflects the demand for iodine by the thyroid gland, reflects longer-term iodine exposure and is more appropriate for assessing status at the individual level [159,160]. Circulating thyroglobulin increases in iodine deficiency, with a cutoff of >13 μg/L suggested for characterizing deficiency in pregnancy [159,160].

### Inflammatory and metabolic indicators

Inflammatory biomarkers CRP and AGP are measured to assess subclinical acute or chronic infections. Specifically, retinol, Hb, ferritin, sTfR, and zinc will be adjusted for inflammation if warranted [149,150].

Clinical biomarkers measured using point-of-care instruments before starting supplementation and during follow-up visits help identify potential hepatic or renal conditions or electrolyte imbalances that could affect a participant's ability to metabolize nutrients. These biomarkers, particularly electrolytes, and minerals, are homeostatically regulated [74,161,162]. Random blood sugar and complete lipid profiles are evaluated both to characterize metabolic risk and, in the case of lipids, to serve as a reference (total cholesterol) against which vitamin E status can be assessed.

### Performance assessment of micronutrient biomarker assays

For the primary outcomes, we provide information on internal performance and external quality assurance processes. The interassay CVs in our study (Supplemental Table 2) are well within the allowable imprecision ranges published by VITAL-EQA performance evaluation program for vitamin A (<2.4%–7.1%), D (<2.8%–8.5%), B12 (<3.4%–10.1%), ferritin (<3.7%–11.2%), and sTfR (<2.8%–8.5%) [163]. We are ensuring the performance of the assays using 3 approaches where available: by validating against NIST SRM, engaging in external laboratory quality assurance schemes [46,47,48], and using independent commercial QC materials with established ranges for particular analytical platforms. NIST-SRMs provide certified values, which are initially used to ensure analyte accuracy within assay systems, as well as running them alongside study samples to monitor assay performance over time. Quality assurance schemes like VITAL-EQA, MVP, and EQUIP by CDC, and proficiency testing by CAP provide guidance on acceptable values and variability, but do not necessarily confirm true accuracy [46,47,48,163] as they rely on pooled QC materials, and compared against routine methods with lower sensitivity and wider uncertainty [163] or peer group means [48], rather than gold-standard methods. An exception is the Vitamin D External Quality Assessment Scheme, which uses SRMs and a gold-standard method, but is limited to a single nutrient [164]. Commercial controls similarly report acceptable ranges for analytes for a given instrument type and, therefore, might ensure comparability across a given platform but not true accuracy. Despite the limitations, these approaches are being utilized in this study to ensure, to the extent possible, the accuracy of our findings. For several primary biomarkers, such as RBP, holoTC, GPX-3, UPLC, and EKA assays for B1, B2, and B3, such quality assurance tools are unavailable but, where possible, we are using published protocols [53,55], identifying experts in the field [165], and using methods with evidence of validation against more established assays [59,61]. In many cases, we are dependent on manufacturer-provided controls and our own house pools, which do not allow for external validation but offer means to monitor assay consistency over time.

### Strengths and limitations

The MiNDR trials include over 30 static and functional biomarkers of micronutrient status across sample types (serum, plasma, whole blood, RBC pellets, urine, and breastmilk), representing one of the most comprehensive biomarker assessments performed in any micronutrient supplementation study to date. However, biomarker-based studies present several challenges, including appropriate selection of biomarkers for comprehensive status assessment, controlling preanalytical conditions, standardization of analytic methods, and availability of appropriate QC materials for evaluating assay performance, as discussed. Biomarker interpretation in conditions like pregnancy and inflammation poses additional challenges due to the lack of appropriate cutoffs relevant to these physiological states. During pregnancy, the interpretation of micronutrient biomarkers is more complex due to the plasma volume expansion (∼6% rise in early gestation and 42%–48% in late pregnancy) [114], increased metabolic demand and placental nutrient transfer to the fetus, and other adaptations. Finally, although women received varying doses of choline, vitamin C, K, pantothenic acid (B5), and biotin, biomarkers for these nutrients were not measured in this study, highlighting the need for future analysis using archived samples.

In conclusion, the MiNDR trials in pregnant and nonpregnant WRA offer a unique opportunity to evaluate the dose–response relationship to increasing doses of micronutrients, using both static and functional biomarkers of status, to determine the optimal amount of nutrients required to achieve sufficiency without increasing the risk of excess. Documentation provided here has broader utility and can serve as a resource for researchers and program implementers designing similar micronutrient intervention trials and programs with biomarkers of status as outcomes. Findings from the MiNDR trials will potentially inform appropriate dosing of micronutrients in MMS products in LMIC settings.

## Author contributions

The authors’ responsibilities were as follows – PC, KJS: conceptualized the study; SBandyo, AKR, Sb, KKS, XG, YW, KJS: outlined the analytical plan, and led laboratory analysis of the micronutrient biomarker assays; TJS, KBA, EZ, HA, RH: led all aspects of study implementation including data and biological sample collection and oversight of all the field activities; LSFW: managed field and laboratory database; SBandyo, XG, EZ, LSFW, BL, MG, PC, KJS: involved in data analysis and interpretation; SMTH: site principle investigator in Bangladesh; SBandyo: wrote the first draft of the manuscript, performed the literature search, and prepared the revised version; PC, KJS: provided edits and revisions to the manuscript; KJS: hold primary responsibility for the final content; and all authors: read and approved the final manuscript.

## Funding

This work was supported by the Bill and Melinda Gates Foundation grant INV033628. The sponsor is not involved in the study design, data collection or management, analysis, or dissemination.

## Conflict of interest

The authors report no conflicts of interest.
