# Supplementary material for: Micronutrient Biomarker Selection and Assay Methods and Performance in Double-Blind, Randomized, Controlled Micronutrient Dose Response (MiNDR) Trials among Women of Reproductive Age and Pregnant Women in Rural Bangladesh
Source: Curr Dev Nutr. 2025 Sep 1;9(10):107546. doi: 10.1016/j.cdnut.2025.107546 (PMC12550162; doi:10.1016/j.cdnut.2025.107546)
Supplement: Multimedia component 1 [file mmc1.docx]

Supplementary Table 1. Micronutrient content of BEP and test products in multiples of recommended intakes by intervention arms^1^

| Nutrient | Total MN by Level + BEP^2^ | | | | Multiples of RDA/AI | | RDA/AI^3^ | UL^4^ |
| --- | --- | --- | --- | --- | --- | --- | --- | --- |
|  | BEP + Level 0 | BEP + Level 1 | BEP + Level 2 | BEP + Level 3 | WRA | PW | WRA/PW | |
| Vitamin A (IU) | 2564 | 3746 | 5661 | 7492 | 1.1-3.2 | 1-2.9 | 2333/2567 | 10000 |
| Vitamin D (IU) | 600 | 1400 | 2200 | 3000 | 1.0-5.0 | 1.0-5.0 | 600 | 4000 |
| Vitamin E (IU)^4^ | 24 | 75 | 186 | 298 | 1.1-13.3 | 1.1-13.3 | 33.3 | 1000 |
| Vitamin B1 (mg) | 1.4 | 2.8 | 4.2 | 5.6 | 1.3-5.1 | 1.0-4.0 | 1.1/1.4 | ND |
| Vitamin B2 (mg) | 1.4 | 2.8 | 4.2 | 5.6 | 1.3-5.1 | 1.0-4.0 | 1.1/1.4 | ND |
| Vitamin B3 (mg)^5^ | 18 | 35 | 65 | 100 | 1.3-7.1 | 1.0-5.6 | 14/18 | ND |
| Vitamin B6 (mg) | 1.9 | 4 | 6 | 10 | 1.5-7.7 | 1.0-5.3 | 1.3/1.9 | 100 |
| Vitamin B9 (µg) | 400 | 400 | 400 | 400 | 1.0 | 0.7 | 400/600 | 1000 |
| Vitamin B12 (µg) | 2.6 | 6 | 12 | 18 | 1.1-7.5 | 1.0-6.9 | 2.4/2.6 | ND |
| Vitamin C (mg) | 100 | 120 | 120 | 120 | 1.3-1.6 | 1.2-1.4 | 75/85 | 2000 |
| Iron (mg) | 30 | 30 | 40 | 40 | 1.7-2.2 | 1.1-1.5 | 18/27 | 45 |
| Zinc (mg) | 15 | 15 | 20 | 20 | 1.9-2.5 | 1.4-1.8 | 8/11 | 40 |
| Iodine (µg) | 220 | 220 | 290 | 290 | 1.5-1.9 | 1-1.3 | 150/220 | 1100 |
| Selenium (µg) | 65 | 100 | 200 | 300 | 1.2-5.5 | 1.1-5.0 | 55/60 | 400 |
| Copper (mg) | 1 | 1 | 1 | 1 | 1.1 | 1.0 | 0.9/1.0 | 10 |
| Choline (mg) | -- | 550 | 750 | 900 | 0-2.1 | 0-2.0 | 425/450^*^ | 3500 |
| Calcium (mg) | 500 | 500 | 500 | 500 | 0.5 | 0.5 | 1000 | 2500 |
| Phosphorus (mg) | 383 | 383 | 383 | 383 | 0.5 | 0.5 | 700 | 4000/3500 |
| Vitamin K (µg) | 90 | 90 | 90 | 90 | 1.0 | 1.0 | 90^*^ | ND |
| Vitamin B5 (mg) | -- | 7 | 7 | 7 | 0-1.4 | 0-1.2 | 5/6^*^ | ND |
| Biotin (µg) | -- | 35 | 35 | 35 | 0-1.2 | 0-1.2 | 30^*^ | ND |
| Potassium (mg) | -- | 1000 | 1000 | 1000 | 0-0.4 | 0-0.3 | 2600/2900^*^ | ND |
| Manganese (mg) | -- | 2.6 | 2.6 | 2.6 | 0-1.4 | 0-1.3 | 1.8/2.0^*^ | 11 |
| Magnesium (mg)^6^ | -- | 145 | 145 | 145 | 0-0.5 | 0-0.4 | 310/350 | 350 |

BEP: balanced energy protein, MN: micronutrient, RDA: recommended dietary allowance, AI: adequate intake, UL: tolerable upper intake level, WRA: women in reproductive age, PW: pregnant woman, ND: not determined.

^1^Intervention arms will receive BEP + MN powder with any of the three levels (1-3) of micronutrient powders, and control arm will receive BEP + placebo powder (level 0) with the same properties but no nutrients.

^2^BEP product contains protein – 14.3g, fat – 21.8g, carbohydrate – 32.2 g (including 9.6 g of sugar), and energy - 382.4 Kcal per serving (75 g)

^3^Dietary reference intakes are reported as AI (^*^) when RDA are not available.

^4^RDA and UL are reported as synthetic forms of α-tocopherol.

^5^Vitamin B3 is provided as niacinamide in the test products. UL for vitamin B3 is based on the levels of nicotinic acid associated with skin flushing and nicotinamide does not cause skin flushing.

^6^RDA considers magnesium from all dietary sources, while UL includes magnesium only from dietary supplements and medications.

Supplementary Table 2. Micronutrient biomarker assay performance for primary outcomes in the MiNDR trials^1^

| **Nutrient** | **Biomarkers** | **Quality control material** | **Reference/certified values**  **Mean±SD/range** | **Inter-assay CV (%)^2^** |
| --- | --- | --- | --- | --- |
| Vitamin A | Retinol | SRM 968f, 2 levels | Level 1: 1.141±0.045 µmol/L  Level 2: 2.30±0.10 µmol/L | Level 1: 3.3  Level 2: 2.0 |
|  | Retinyl palmitate | SRM 968f, 2 levels (consensus values) | Level 1:0.017±0.015 µg/mL  Level 2: 0.030±0.029 µg/mL | Level 1: 17.1  Level 2: 8.9 |
|  | RBP | Manufacturer provided, 3 levels; Quantikine Immunoassay, R&D systems | Level 1: 7.89-12.9 µg/mL Level 2: 20.6-33.6 µg/mL  Level 3: 41.0-66.9 µg/mL | Level 1: 4.5 Level 2: 6.4  Level 2: 6.8 |
| Vitamin D | 25(OH)D^3^ | Manufacturer provided, 2 levels;  PreciControl, Roche Diagnostics | Level 1: 54.3 (36.4-72.2) nmol/L Level 2: 97.8 (65.5-127) nmol/L | Level 1: 6.5  Level 2: 5.9 |
| Vitamin E | α-tocopherol | NIST SRM 968f, 2 levels | Level 1: 11.95±0.49 µmol/L  Level 2: 27.5±1.7 µmol/L | Level 1: 7.1  Level 2: 4.9 |
|  | γ-tocopherol | NIST SRM 968f, 2 levels | Level 1: 2.63±0.12 µmol/L  Level 2: 6.21±0.34 µmol/L | Level 1: 9.4  Level 2: 9.9 |
| Vitamin B_6_  (Pyridoxin) | PLP | SRM 3950, 2 levels | Level 1: 18.6±0.6 nmol/L  Level 2: 36.4±1.2 nmol/L | Level 1: 11.0  Level 2: 4.1 |
|  | 4-PA | NIST SRM 3950, 2 levels (values of potential interest) | Level 1: 121 nmol/L  Level 2: 202 nmol/L | Level 1: 6.9  Level 2: 8.4 |
| Vitamin B_12_ | Total cobalamin^3,4^ | Manufacturer provided, 2 levels, Eleesys PreciControl, Roche Diagnostics | Level 1: 482 (323-641) pg/mL Level 2: 932 (569-1295) pg/mL | Level 1: 5.2 Level 2: 4.3 |
|  | HoloTC | Manufacturer provided, 2 levels, Tecan, IBL International GmbH; in-house pools | Level 1: 23.8 (15-35) pmol/L Level 2: 61.7 (36-84) pmol/L | Level 1: 3.5 Level 2: 2.5 |
|  | Homocysteine | Commercial controls, 3 levels, Lyphocheck,  Bio-Rad Laboratories Inc. | Level 1: 6.68 (3.9-9.4) µmol/L Level 2: 12.3 (8.1-16.5) µmol/L  Level 3: 27.5 (19.3-35.6) µmol/L | Level 1: 8.3 Level 2: 4.9  Level 3: 4.2 |
| Iron | Ferritin^3,4^ | Manufacturer provided, 2 levels; Eleesys PreciControl Varia Level 1 and 2 (Roche Diagnostics GmbH) | Level 1: 140 (102-178) ng/mL Level 2: 852 (622-1082) ng/mL | Level 1: 5.8  Level 2: 5.3 |
|  | STfR^3^ | Manufacturer provided, 2 levels (Roche Diagnostic GmbH) | Level 1: 2.29 (1.96-2.62) mg/L Level 2: 7.34 (6.23-8.45) mg/L | Level 1: 5.5  Level 2: 4.4 |
| Selenium | Serum selenium | SRM 1598a, 1 level  Commercial controls, 2 levels, Seronorm Trace Elements Serum, SERO | NIST: 134.4 ± 5.8 µg/L  Seronorm level 1: 95 ± 19 µg/L  Seronorm level 2: 139 ± 28 µg/L | NIST: 8.3  Level 1: 8.1  Level 2: 8.0 |
| Zinc | Serum zinc | SRM 1598a, 1 level  Commercial controls, 2 levels, Seronorm Trace Elements Serum, SERO | NIST: 880 ± 24 µg/L  Seronorm level 1: 1460 ± 290 µg/L  Seronorm level 2: 2090 ± 315 µg/L | NIST: 12.3  Level 1: 7.5  Level 2: 7.6 |
| Iodine | Urinary iodine^5^ | EQUIP samples, Level 1, 2, and 3 | Level 1: 60.1 (45.1-75.1) µg/L  Level 2: 155.9 (124.7 - 187.1) µg/L  Level 3: 345.6 (293.8 - 397.4) µg/L | Level 1: 11.0 Level 2: 9.2  Level 3: 6.2 |
|  | Thyroglobulin | Commercial controls, 3 levels, Lyphocheck,  Bio-Rad Laboratories Inc. | Level 1: 6.0 (3.6-8.5) ng/mL Level 2: 51.7 (37.4-66.1) ng/mL  Level 3: 148 (109-187) ng/mL | Level 1: 2.5 Level 2: 3.5  Level 3: 1.8 |

Abbreviations: EQUIP, Ensuring the Quality of Urinary Iodine Procedures; Holo-TC, holotranscobalamin; 25(OH)D, 25-hydroxy vitamin D; NIST, National Institute of Standards and Technology; PLP, pyridoxal 5’-phosphate; 4-PA, 4-pyridoxic acid; RBP, retinol binding protein; sTfR, soluble transferrin receptor; SRM, standard reference material

^1^Standardization of assays is underway for biomarkers of vitamin B1 (thiamin excretion, erythrocyte transketolase activity), vitamin B2 (riboflavin excretion and erythrocyte glutathione reductase activity), and vitamin B3 (niacin excretion metabolites), functional assays for selenium (glutathione peroxidase-3), and iodine (thyroglobulin).

^2^The inter-assay CV is calculated as the standard deviation of the five daily mean QC values divided by the mean concentration, expressed as a percentage.

^3^Assay performance evaluated through Vitamin A Laboratory – External Quality Assurance (VITAL-EQA) (1), and Micronutrients Performance Verification (MPV) programs by CDC (2).

^4^Assay performance evaluated through Laboratory Accreditation Program by College of American Pathologists program (CAP) (3).

^5^Assay performance evaluated through Ensuring the Quality of Urinary Iodine Procedures (EQUIP) by CDC (4).

**References:**

1. Centers for Disease Control and Prevention (CDC). Vitamin A Laboratory – External Quality Assurance (VITAL-EQA) [Internet]. Available from: https://www.cdc.gov/laboratory-quality-assurance/php/nutritional-biomarkers/vital-eqa.html

2. Centers for Disease Control and Prevention (CDC). Performance Verification Program for Serum Micronutrients [Internet]. Available from: https://www.cdc.gov/laboratory-quality-assurance/php/nutritional-biomarkers/serum-micronutrients-performance.html

3. College of American Pathologists (CAP). Laboratory Accreditation Program [Internet]. Available from: https://documents.cap.org/documents/CLIA-table-for-cap.org-3.pdf

4. Centers for Disease Control and Prevention (CDC). Ensuring the Quality of Urinary Iodine Procedures (EQUIP) [Internet]. Available from: https://www.cdc.gov/laboratory-quality-assurance/php/inorganic-elements/equip.html#:~:text=Ensuring%20the%20Quality%20of%20Iodine,in%20more%20than%2090%20countries.
